# Brazilian caregiver version of the Apathy Scale

**DOI:** 10.1590/S1980-57642009DN30400010

**Published:** 2009

**Authors:** Henrique Cerqueira Guimarães, Patricia Paes Araujo Fialho, Viviane Amaral Carvalho, Etelvina Lucas dos Santos, Paulo Caramelli

**Affiliations:** 1 Behavioral and Cognitive Neurology Unit, Department of Internal Medicine, Faculty of Medicine of the Federal University of Minas Gerais, Belo Horizonte MG, Brazil.; 2 Post-graduate Program in Neurology, University of São Paulo School of Medicine, São Paulo SP, Brazil.

**Keywords:** apathy, diagnosis, evaluation, dementia, Alzheimer’s disease

## Abstract

No Brazilian version of a specific scale for evaluating apathy in dementia is available.

**Objectives:**

To introduce a translated version of the Apathy Scale (AS) for use with caregivers.

**Methods:**

The instrument was formally translated and then administered to the caregivers of a small sample of dementia patients, in order to assess scale comprehensibility and make final adjustments. The scale was subsequently administered to the caregivers of a second, independent sample of Alzheimer’s disease (AD) patients. The content validity of the scale was tested by correlating the AS scores with the Neuropsychiatric Inventory (NPI) - apathy sub-score and Disability Assessment in Dementia (DAD) total scores.

**Results:**

The first sample consisted of eleven subjects with dementia, most of whom had AD. The second sample comprised twenty patients with probable or possible AD (10 with mild dementia), a mean age of 84.1±5.8 years, and 2.2±1.6 years of schooling. The AS scores correlated with both NPI-apathy sub-score (r=0.756, p=0.001) and DAD total scores (r=–0.793, p=0.0005).

**Conclusions:**

The final version had good comprehensibility and correlated strongly with standardized apathy and functional activities of daily living measures.

## Abstract (pt)

Não há versão brasileira de qualquer escala especificamente dedicada à avaliação de apatia nas demências.

**Objetivos:**

Apresentar uma versão traduzida da Escala de Apatia direcionada ao cuidador.

**Métodos:**

O instrumento foi formalmente traduzido e administrado aos cuidadores de uma pequena amostra de pacientes com demência, para avaliar a compreensibilidade da versão e realizar ajustes finais. Em seguida a escala foi administrada aos cuidadores de uma segunda amostra independente de pacientes com doença de Alzheimer (DA). A validade de conteúdo da escala foi testada correlacionando-se os escores da Escala de Apatia com os subescores de apatia do Inventário Neuropsiquiátrico (NPI) e com os escores totais da escala DAD de incapacidade na demência.

**Resultados:**

A primeira amostra consistia de onze pacientes com demência, a maioria com doença de Alzheimer (DA). A segunda amostra possuía vinte pacientes com DA provável ou possível (10 com demência leve), média de idade de 84,1±5,8 anos e escolaridade média de 2,2±1,6 anos. Os escores na Escala de Apatia correlacionaram-se com o subescore apatia do NPI (r=0,756; p=0,001) e com os escores totais da DAD (r=–0,793; p=0,0005).

**Conclusões:**

A versão final apresentou boa compreensibilidade e correlacionou-se fortemente com medidas padronizadas de apatia e atividades de vida diária.

Apathy was initially defined by Marin^1^ as “lack of motivation, relative to the patient’s previous level of functioning or the standards of his or her age and culture, not attributable to intellectual impairment, emotional distress or diminished level of consciousness.”

There is a fairly strong consensus in the literature that apathy should be considered a separate syndrome in dementia, with specific clinical and prognostic implications.^2,3^ Apathy has been consistently shown to be the most prevalent neuropsychiatric disorder in dementia,^4^ especially in the context of Alzheimer’s disease (AD),^5^ where apathy has been associated with worse executive functioning^6,7^ and more severe extrapyramidal signs.^8^ Additionally, a prospective study has shown that the emergence of apathy in a cohort of AD patients was significantly associated with worse cognitive and functional performance in follow-up.^9^

Another important feature regarding apathy evaluation pertains to its distinction from depression.^10^ Since Marin’s initiative,^11^ several other authors have proposed specific instruments to evaluate and quantify this syndrome in dementia,^12-16^ helping to unravel this issue. To date, the most widely used instrument in the literature is the Neuropsychiatric Inventory (NPI).^17^ However, this tool has several limitations: it is not specifically dedicated to evaluate apathy; it identifies non-relevant clinical symptoms when apathy scores fall below four; and lastly, since the NPI relies on a screening question, and apathy seems to be a heterogeneous disorder, some definite apathetic patients can be missed by the screening if the examiner is inexperienced.

Most of the research on apathy associated with AD can be ascribed to Starkstein’s and his coworker’s.^2,8,9^ Using the Apathy Scale (AS)^12^ – an instrument with 14 questions adapted from Marin’s original 18-item Apathy Evaluation Scale (AES) – their group showed that apathy has major prognostic implications in AD. The scores on the AS range from zero to 42 points, with higher scores indicating greater severity of symptoms.

Based on the auspicious work of Starkstein et al. we believe it is time to improve our diagnostic capabilities and to better characterize apathy phenomenology. Additionally, any trial investigating interventions aimed at improving apathetic symptoms must have primary efficacy measures analyzed by instruments specifically dedicated to evaluating apathy in dementia. An ideal apathy quantifying scale should have a wide range of possible scores and should also be brief and easy to administer. We believe that the AS meets most of these requirements.

Although we already have a version of the NPI in Brazil,^18^ we are unaware of a specific scale for evaluating apathy in our country. The primary aim of this study was to introduce a Portuguese version of the AS, suitable for caregiver interview, and to describe some of its basic and preliminary psychometric properties.

## Methods

The study was conducted in three phases. In the first phase, the original version of the AS was translated independently by two of the researchers (HCG and PC). A consensus was reached to define the final translated version, which was then back translated by a linguistic expert. The back translated version was compared with the original for final adjustments. Minor adaptations to the final version were necessary in order to make it suitable for caregiver interview. Briefly, we only changed the questions from first to third person.

In the second phase, the final translated version was used to interview the caregivers of a small sample of 11 patients with AD or frontotemporal dementia (FTD). This pilot stage was designed to assess the translated scale’s comprehensibility, and make any necessary adjustments to the instrument. The subjects were recruited from the Behavioral and Cognitive Neurology Outpatient Unit at the Hospital das Clínicas from the Federal University of Minas Gerais in Belo Horizonte (MG), Brazil. Caregivers were defined as those who spent most time with the patient, usually on a daily basis, and at least 12 hours a week. The study was approved by the local ethics committee and all participants gave their written informed consent.

In the third phase, another independent sample consisting of 20 patients fulfilling the diagnosis of either possible or probable AD were randomly selected from among the demented subjects identified in a large population-based epidemiological survey, The PIETÀ study,^19^ conducted in Caeté, Minas Gerais state, southeast Brazil. This study also has local ethics committee approval and all the participants gave written informed consent. To form this sample, 10 patients were selected with mild stage dementia and ten with moderate or moderate advanced stages, according to the Functional Assessment Staging in Alzheimer’s disease.^20^ All the subjects were evaluated with the Mini-Mental State Examination.^21^ Caregivers were defined as outlined above and were submitted to an interview consisting of the AS, NPI^17,18^ and Disability Assessment for Dementia (DAD).^22,23^

Dementia diagnosis was established according to DSM-IV criteria (APA).^24^ AD and FTD were diagnosed according to standard published criteria.^25,26^ AS was always administered by the same examiner (HCG); the other evaluations were administered by experienced neuropsychologists (ELS, PPAF and VAC). For statistical analysis, Spearman’s rank correlation tests were performed between AS, NPI – apathy and DAD scores. The significance level adopted was 0.05.

## Results

The original^12^ and the final version of the translated scale are shown in the Appendix at the end of the manuscript.

In the pilot phase, the first sample consisted of 11 patients, whose caregivers were interviewed with the translated scale. Eight of the patients had AD (four women), a mean age of 73.8±4.7 years and mean educational level of 5.8±4.2 years, all presenting mild stage of dementia (FAST 4). The remaining three patients had FTD (two women), were aged 55.0±8.7 years and had 10.0±6.6 years of schooling. Based on clinical judgment it seemed that two of the FTD subjects were at mild stages of dementia (subjects 10 and 11), since there is no standard method for staging this kind of patient.

All caregivers exhibited good comprehension of the instrument. No final adjustments were necessary after this pilot study. On average, it took around ten minutes to complete the scale. For this first sample, mean scores on the AS were 22.8±8.4 points.

The second sample was composed by the 20 patients with probable or possible AD, 10 at a mild dementia stage (FAST=4) and 10 at moderate or moderate advanced dementia stages (FAST=5 or 6). There were 14 women and six men, aged 84.1±5.8 years, with a mean of 2.2±1.6 years of schooling. The mean performance on the Mini-Mental State Examination (MMSE) was 17.4±4.7, reflecting the low schooling of the sample, even though half of the patients had mild dementia. The caregivers were predominantly women (90%), most of them daughters (80%), aged 55.2±11.6 years, with a mean of 9.7±4.1 years of schooling. There was missing age data for two of the caregivers and schooling data was lacking for another. For this second sample, mean scores on the AS were 23.6±10.6(range: 9-40), NPI-apathy sub-scores were 4.2±4.4(range: 0-12) and DAD total scores were 25.4±7.3(range: 8-37).

**Figure 1:**
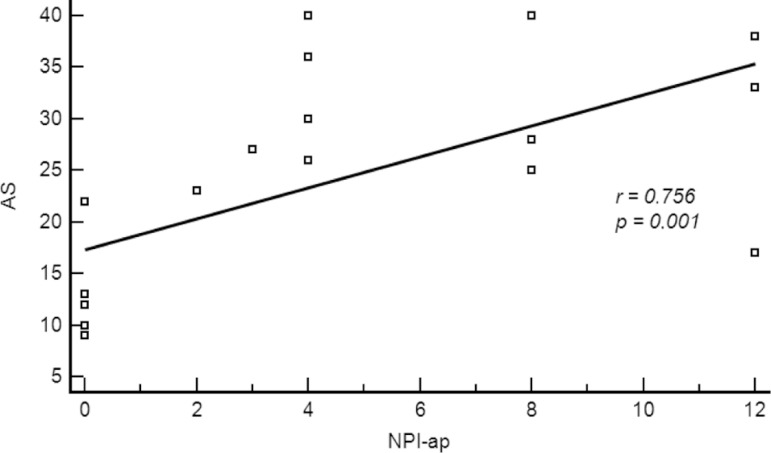
Correlation between Apathy Scale (AS) scores and Neuropsychiatric Inventory apathy (NPI-ap) sub-scores.

**Figure 2:**
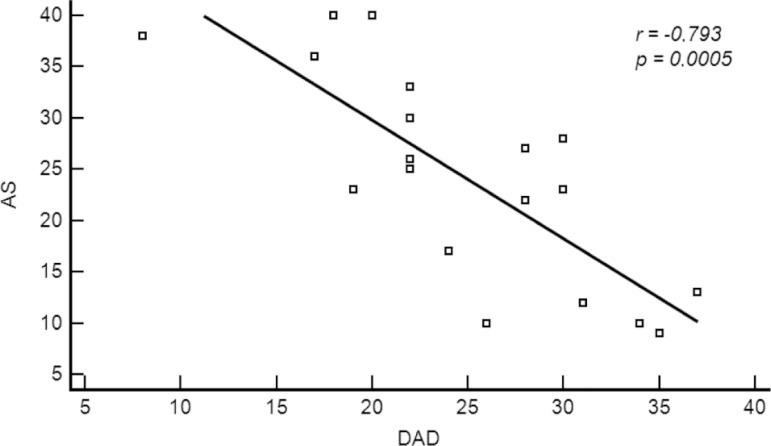
Correlation between Apathy Scale (AS) scores and Disability Assessment for Dementia (DAD) total scores.

**Figure 3:**
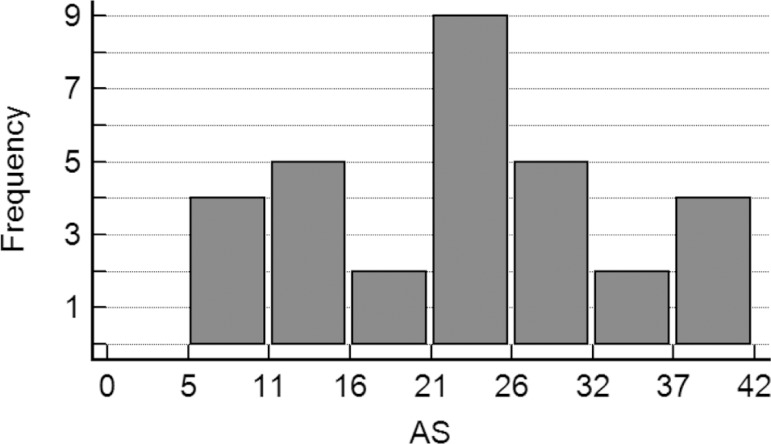
Histogram showing the distribution of 31 Apathy Scale (AS) scores from both samples.

In the second AD patient sample, the AS scores correlated strongly with the NPI-apathy sub-score (r=0.756, p=0.001; Figure 1). Additionally, we found a robust inverse correlation between AS and performance on activities of daily living assessed with the DAD (r=–0.793, p=0.0005; Figure 2). We also found a moderate correlation between AS scores and FAST categories from the 28 AD patients from both first and second samples (r=0.401, p=0.037). We conducted additional analysis and found no correlation between NPI-depression sub-scores or any of the presented variables (data not shown). Finally, we grouped together all patients from the two study phases in order to compare the distribution of the 31 AS scores (Figure 3). The histogram shows a fairly wide range of results, at least in this study in which subjects at mild dementia stages predominated.

## Discussion

In general, the interviewed caregivers displayed good comprehension for all the questions of the AS. Clarifications were sometimes necessary, especially regarding quantitative issues, such as “interests”. In this case, we usually attained to the scale question and instructed the caregiver to compare the number of current interests with the ones the patient had before the memory impairment was noticed. In a few instances, caregivers with low schooling needed a brief explanation of what “apathetic” meant. In this case, a broad definition such as “uninterested, unmotivated, indifferent and unconcerned” was used.

The principal strength of this study is the confirmation of content validity for the translated version. Strong correlations were found between AS, NPI-apathy and DAD scores. Although the NPI is the most widely used tool for quantifying apathy in dementia research, it also has significant limitations and is heavily dependent on examiner experience.^27^ In contrast, DAD is a functional scale for assessment of activities of daily living. It does not seem to depend on examiner expertise and has little influence from subjective bias. The robust correlation between AS and DAD clearly shows that we were measuring clinically meaningful behavioral disorder. Furthermore, depressive symptoms assessed with the NPI do not to explain the above findings.

It seemed that AS fulfilled most of the expected requirements. There is little doubt that this tool measures apathetic symptoms. In most cases, it took on average no more than 10 minutes to complete the scale and a wide range of scores were observed in the studied population. An obvious limitation of this study is that we did not present several important psychometric properties from the scale, such as inter-rater and test retest reliabilities. In response to growing calls from the Brazilian research community in Cognitive Neurology for a Portuguese version of a specific scale for evaluating apathy in dementia, we decided to publish our version in this preliminary paper prior to formal validation.

## Figures and Tables

**Table 1:** Main sociodemographic and clinical characteristics of the first patient sample.

Subject	Gender	Age	Education*	Diagnosis	MMSE	AS
1	male	68	6	AD	19	23
2	fem	78	4	AD	22	19
3	fem	79	4	AD	20	12
4	fem	67	4	AD	16	12
5	male	70	4	AD	23	21
6	male	75	16	AD	24	26
7	male	76	4	AD	23	30
8	fem	77	4	AD	15	25
9	fem	60	11	FTD	17	39
10	male	45	16	FTD	28	14
11	fem	60	3	FTD	20	30

MMSE, Mini-Mental State Examination; AS, Apathy Scale; AD, Alzheimer's disease; FTD, Frontotemporal dementia;

* Formal education in years.

**Table 2:** Main sociodemographic and clinical characteristics of the second patient sample and respective caregiver sample*.

Subject		Caregiver					Patient							
		Gender	Age	Relationship^+^	Educ.^§^		Gender	Age	Educ.^§^	FAST	MMSE	AS	NPI-ap	DAD
1		fem	79	wife	4		male	83	4	4	24	30	4	22
2		fem	51	daughter	13		fem	84	1	4	22	10	0	34
3		fem	33	daughter	13		fem	76	0	4	13	12	0	31
4		fem	59	daughter	11		fem	81	4	4	24	28	8	30
5		fem	38	daughter	15		fem	78	4	4	23	13	0	37
6		fem	62	daughter	8		fem	81	1	4	20	26	4	22
7		fem	56	daughter	11		fem	79	4	4	15	27	3	28
8		fem	51	daughter	4		fem	87	0	4	16	9	0	35
9		fem	58	daughter	NA		fem	90	4	4	23	10	0	34
10		fem	67	daughter	4		fem	93	3	4	13	25	8	22
11		fem	58	daughter	15		male	84	4	5	21	40	8	20
12		male	52	son	11		male	86	0	5	17	23	2	30
13		fem	72	wife	3		male	75	1	5	14	40	4	18
14		fem	56	daughter	11		fem	96	2	5	9	23	2	19
15		fem	60	daughter	14		fem	88	2	5	15	22	0	28
16		male	53	son	11		fem	78	1	5	15	17	12	24
17		fem	NA	granddaughter	11		male	92	4	5	21	10	0	26
18		fem	37	daughter	13		male	87	0	6	10	33	12	22
19		fem	51	other	9		fem	82	1	6	19	36	4	17
20		fem	NA	daughter	4		fem	81	3	6	14	38	12	8

FAST, Functional Assessment Staging; MMSE, Mini-Mental State Examination; AS, Apathy Scale; NPI-ap, Neuropsychiatric Inventory - apathy sub-score; DAD, Disability Assessment for Dementia total score; NA, data not available;

* All subjects fulfilled diagnosis of either possible or probable Alzheimer's disease;

+ familial relationship;

§ formal education in years

## APPENDIX 1

**Table:** Original version of the Apathy Scale^12^

1. Are you interested in learning new things?	not at all (3)	slightly (2)	some (1)	a lot (0)
2. Does anything interest you?	not at all (3)	slightly (2)	some (1)	a lot (0)
3. Are you concerned about your condition?	not at all (3)	slightly (2)	some (1)	a lot (0)
4. Do you put much effort into things?	not at all (3)	slightly (2)	some (1)	a lot (0)
5. Are you always looking for something to do?	not at all (3)	slightly (2)	some (1)	a lot (0)
6. Do you have plans and goals for the future?	not at all (3)	slightly (2)	some (1)	a lot (0)
7. Do you have motivation?	not at all (3)	slightly (2)	some (1)	a lot (0)
8. Do you have the energy for daily activities?	not at all (3)	slightly (2)	some (1)	a lot (0)
9. Does someone have to tell you what to do each day?	not at all (3)	slightly (2)	some (1)	a lot (0)
10. Are you indifferent to things?	not at all (3)	slightly (2)	some (1)	a lot (0)
11. Are you unconcerned with many things?	not at all (3)	slightly (2)	some (1)	a lot (0)
12. Do you need a push to get started on things?	not at all (3)	slightly (2)	some (1)	a lot (0)
13. Are you neither happy nor sad, just in between?	not at all (3)	slightly (2)	some (1)	a lot (0)
14. Would you consider yourself apathetic?	not at all (3)	slightly (2)	some (1)	a lot (0)
Total (0-42)				

## APPENDIX 2

**Table:** Brazilian caregiver version of the Apathy Scale

1. Ele/ela está interessado em aprender coisas novas?	de jeito nenhum (3)	um pouco (2 )	mais ou menos (1)	muito(0)
2. Há alguma coisa que interesse a ele/ela?	de jeito nenhum (3)	um pouco (2 )	mais ou menos (1)	muito(0)
3. Ele/ela aparenta estar preocupado(a) com a sua condição?	de jeito nenhum (3)	um pouco (2 )	mais ou menos (1)	muito(0)
4. Ele(a) se esforça nas coisas que faz?	de jeito nenhum (3)	um pouco (2 )	mais ou menos (1)	muito(0)
5. Ele(a) está sempre procurando alguma coisa para fazer?	de jeito nenhum (3)	um pouco (2 )	mais ou menos (1)	muito(0)
6. Ele/ela tem planos ou metas para o futuro?	de jeito nenhum (3)	um pouco (2 )	mais ou menos (1)	muito(0)
7. Ele/ela tem motivação?	de jeito nenhum (3)	um pouco (2 )	mais ou menos (1)	muito(0)
8. Ele/ela tem disposição para as atividades diárias?	de jeito nenhum (3)	um pouco (2 )	mais ou menos (1)	muito(0)
9. Alguém tem que dizer a ele/ela o que fazer a cada dia?	de jeito nenhum (3)	um pouco (2 )	mais ou menos (1)	muito(0)
10. Ele(a) está indiferente às coisas?	de jeito nenhum (3)	um pouco (2 )	mais ou menos (1)	muito(0)
11. Ele/ela está despreocupado(a) com muitas das coisas?	de jeito nenhum (3)	um pouco (2 )	mais ou menos (1)	muito(0)
12. Ele/ela necessita de um empurrão para iniciar as coisas?	de jeito nenhum (3)	um pouco (2 )	mais ou menos (1)	muito(0)
13. Ele /ela aparenta estar nem feliz nem triste, simplesmente no meio termo?	de jeito nenhum (3)	um pouco (2 )	mais ou menos (1)	muito(0)
14. Você o(a) considera apático?	de jeito nenhum (3)	um pouco (2 )	mais ou menos (1)	muito(0)
Total (0-42):				
