# Erratum

**Published:** 2010

**Brazilian caregiver version of the Apathy Scale**

In the paper entitled “Brazilian caregiver version of the Apathy Scale” by Guimarães et al. ( (Dement Neuropsychol 2009;3 (4):321-326), there was a mistake in the scores of the apathy scale. The correct original and Brazilian versions are depicted below:

**APPENDIX 1:** Original version of the Apathy Scale^12^

1. Are you interested in learning new things?	not at all (3)	slightly (2)	some (1)	a lot (0)
2. Does anything interest you?	not at all (3)	slightly (2)	some (1)	a lot (0)
3. Are you concerned about your condition?	not at all (3)	slightly (2)	some (1)	a lot (0)
4. Do you put much effort into things?	not at all (3)	slightly (2)	some (1)	a lot (0)
5. Are you always looking for something to do?	not at all (3)	slightly (2)	some (1)	a lot (0)
6. Do you have plans and goals for the future?	not at all (3)	slightly (2)	some (1)	a lot (0)
7. Do you have motivation?	not at all (3)	slightly (2)	some (1)	a lot (0)
8. Do you have the energy for daily activities?	not at all (3)	slightly (2)	some (1)	a lot (0)
9. Does someone have to tell you what to do each day?	not at all (0)	slightly (1)	some (2)	a lot (3)
10. Are you indifferent to things?	not at all (0)	slightly (1)	some (2)	a lot (3)
11. Are you unconcerned with many things?	not at all (0)	slightly (1)	some (2)	a lot (3)
12. Do you need a push to get started on things?	not at all (0)	slightly (1)	some (2)	a lot (3)
13. Are you neither happy nor sad, just in between?	not at all (0)	slightly (1)	some (2)	a lot (3)
14. Would you consider yourself apathetic?	not at all (0)	slightly (1)	some (2)	a lot (3)
Total (0-42)				

**APPENDIX 2:** Brazilian caregiver version of the Apathy Scale

1. Ele/ela está interessado em aprender coisas novas?	de jeito nenhum (3)	um pouco (2)	mais ou menos (1)	muito (0)
2. Há alguma coisa que interesse a ele/ela?	de jeito nenhum (3)	um pouco (2)	mais ou menos (1)	muito (0)
3. Ele/ela aparenta estar preocupado (a) com a sua condição?	de jeito nenhum (3)	um pouco (2)	mais ou menos (1)	muito (0)
4. Ele (a) se esforça nas coisas que faz?	de jeito nenhum (3)	um pouco (2)	mais ou menos (1)	muito (0)
5. Ele (a) está sempre procurando alguma coisa para fazer?	de jeito nenhum (3)	um pouco (2)	mais ou menos (1)	muito (0)
6. Ele/ela tem planos ou metas para o futuro?	de jeito nenhum (3)	um pouco (2)	mais ou menos (1)	muito (0)
7. Ele/ela tem motivação?	de jeito nenhum (3)	um pouco (2)	mais ou menos (1)	muito (0)
8. Ele/ela tem disposição para as atividades diárias?	de jeito nenhum (3)	um pouco (2)	mais ou menos (1)	muito (0)
9. Alguém tem que dizer a ele/ela o que fazer a cada dia?	de jeito nenhum (0)	um pouco (1)	mais ou menos (2)	muito (3)
10. Ele (a) está indiferente às coisas?	de jeito nenhum (0)	um pouco (1)	mais ou menos (2)	muito (3)
11. Ele/ela está despreocupado (a) com muitas das coisas?	de jeito nenhum (0)	um pouco (1)	mais ou menos (2)	muito (3)
12. Ele/ela necessita de um empurrão para iniciar as coisas?	de jeito nenhum (0)	um pouco (1)	mais ou menos (2)	muito (3)
13. Ele /ela aparenta estar nem feliz nem triste, simples­mente no meio termo?	de jeito nenhum (0)	um pouco (1)	mais ou menos (2)	muito (3)
14. Você o (a) considera apático?	de jeito nenhum (0)	um pouco (1)	mais ou menos (2)	muito (3)
Total (0-42)				

